# Navigating your US bioscience career into the 2030s

**DOI:** 10.1371/journal.pbio.3003089

**Published:** 2025-03-28

**Authors:** Peter J. Hotez

**Affiliations:** 1 Texas Children’s Hospital Center for Vaccine Development, National School of Tropical Medicine, Baylor College of Medicine, Houston, Texas, United States of America; 2 Department of Biology, Baylor University, Waco, Texas, United States of America; 3 Hagler Institute for Advanced Studies at Texas A&M University, College Station, Texas, United States of America; 4 Scowcroft Institute of International Studies, Bush School of Government and Public Service, Texas A&M University, College Station, Texas, United States of America; 5 James A Baker III Institute of Public Policy, Rice University, Houston, Texas, United States of America; Fred Hutchinson Cancer Research Center, UNITED STATES OF AMERICA

## Abstract

The coming decade might see major cuts to US Government funding for biomedicine and the mainstreaming of pseudoscience. This Perspective highlights how your biosciences PhD gives you the problem-solving skills to navigate this maelstrom, especially if you maintain flexibility, optimism, and enthusiasm for uncharted paths.

## Teaser

The coming decade might see major cuts to the United States Government funding for biomedicine and the mainstreaming of pseudoscience. But your biosciences PhD gives you the problem-solving skills to navigate this maelstrom, especially if you maintain flexibility, optimism, and enthusiasm for uncharted paths.

The pursuit of a meaningful and productive doctoral degree and career in bioscience hasn’t been easy of late. New findings point to: (1) the excessive time it takes to complete doctoral studies—with time-to-degree estimates of 5–7 years; (2) prolonged postdoctoral training or delayed opportunities for independent careers; and (3) limited numbers of academic research positions relative to the supply of PhD-trained scientists [1]. Despite such hurdles, for many scientists the exciting pace of scientific discovery in new biomedical technologies, e.g., synthetic biology, gene editing, precision medicine, etc. (to name a few), still outweigh the negatives. However, the new United States (US) administration has just thrown us two curveballs. Together, they could reduce career prospects for young and mid-career scientists.

The first is a guidance document from the National Institute of Health (NIH) announcing significant reductions in indirect costs provided to institutions receiving federal grants [2]. If implemented, it would result in billions of dollars of diminished support for biomedical research, and cause thousands of highly skilled employees to be let go in the coming months. Fortunately, multiple legal challenges are in place to slow or halt this from happening, but even then, we must also recognize that we can no longer rely on the US federal government to support major growth in biomedical science. The second follows from the appointment of new leadership at the Department of Health and Human Service, giving credence to the antivaccine movement, as well as the “Make America Healthy Again” movement, aimed at disrupting public health, promoting libertarian principles, and prioritizing the wellness industry and its influencers [3].

In the coming years, scientists may therefore face a one-two punch: Declining governmental support for biomedicine and possibly a commensurate rise in pseudoscience. An added concern is the potential downstream impact or knock-on effect on graduate school admissions, with some distinguished graduate programs already dialing back on their programs, or at least for now. Also, compounding this situation is a climate of fear at many research medical schools, as academic leaders worry about US Government retribution to their institutions. A new culture of silence could further enable an already toxic anti-science maelstrom.

While the specter of a maelstrom may seem apocalyptic, the reality is that all is not lost and there remain multiple and interesting options for a meaningful and fulfilling career in bioscience. But navigating this landscape will require thoughtfulness and planning.

First, recognize that your PhD in bioscience or related disciplines provides you with an extraordinary skill set—data analysis, critical thinking, project management, problem solving, and an ability to communicate complex concepts and data. Think of your PhD less in terms of the subject content of your dissertation and more as a well-recognized credential for your mastery of conquering intellectual mountains. You are a powerful thinker, and the proposed NIH cuts are a reminder of something you should have figured out anyway—your value goes way beyond what an NIH study section thinks about your ideas or how you express them.

Second, embrace change and plan accordingly. The declines in governmental support for science linked to an ascending pseudoscience agenda may not be temporary. The anti-science sphere may endure. So “hunkering down” for a couple of years with the expectation we’ll soon return to earlier ways of doing science may not be wise.

Yet, if you have an openness for new adventures there is some good news: Even if NIH budgets shrink or become inadequate to maintain yesterday’s infrastructure, some analytics groups expect private investments in life sciences to remain robust, reaching more than $30 billion in 2025 and possibly doubling before the end of this decade [4]. Much of this growth will cluster in urban life sciences hubs, bringing together universities and research institutes, biotech start-ups and established companies, cadres of management, financial, and clinical trials consultants, substantial venture capital, connections to private equity, and an ecosystem that supports scientific collaboration between these entities [5]. For decades, major life sciences clusters have been in places such as Boston-Cambridge, San Francisco Bay Area, San Diego-La Jolla, and Maryland-Virginia-Washington DC, but now they are also emerging in other areas, including the Research Triangle (North Carolina), the Texas Medical Center (Houston), Chicago, Cleveland … the list goes on.

However, you will need help. Specifically, your successes in life science clusters will require significant culture and infrastructure shifts, ones that would allow unprecedented fluidity between academia, biotech, and the private equity required to support both enterprises. In an ideal world, such a system might encourage you to become a scientist or even professor with options that let you keep one foot in academia, another in industry, and maybe even a third limb in financing. As a laboratory head, your students and postdoctoral fellows must be afforded similar opportunities. Currently, onerous conflict of interest rules and other forces inhibits an easy exchange between academia-biotech-private equity, so it may be important to revisit such restrictions. In these times of government austerity and cutbacks, principal investigators, staff scientists, and doctoral students will require greater freedom to shuttle back and forth between different types of for-profit and non-profit research organizations. In a 2025 interview with *Harvard Magazine*, the eminent biologist Doug Melton, lamented how it is still often not possible to educate students in industry settings, or for “universities [to] partner with the private sector in the education of students seeking advanced degrees, which would require assent and cooperation from both directions” [6].

It is worth pointing out that many of these restrictions are in place to avoid conflicts between federally funded research, ostensibly for the public good, and for-profit companies. Often, intellectual property is at stake. There are multiple examples of scientists who have navigated this successfully and have founded dozens of successful startups. Beyond those mentioned in the article highlighted above, one could look to important scientists such as George Church at Harvard Medical School and MIT, or David Baker at the University of Washington, among others. Recognizing that the federal government is no longer a reliable source of all funding will require universities and research institutes to adopt a more flexible approach and implement safeguards. Indeed, there may be no choice in the matter. Along those lines we might reexamine how we conduct PhD doctoral training and make it nimbler or better suited for biotech or industry. While a 5–7-year PhD under the guidance of a principal investigator to pursue a dissertation and publications in high impact journals still has merit, there should be consideration for an alternative 4-year PhD with a series of 6–9-month projects conducted in both university and industry settings. Given the possibility that traditional NIH T32 training grant funding might also soon diminish, a V.2.0 biotech PhD might offer a means to ensure a future pipeline of well-trained life scientists. Some interesting examples have already begun, such as the 3-year (and fully funded) venture science doctorate provided by the Deep Science Ventures College at Woolf (https://www.deepscienceventures.com/venture-science-doctorate), based in the United Kingdom.

Two final considerations include (1) the necessity of being both deliberate and strategic and (2) the importance of being flexible and even whimsical about your future.

Regarding the first point, even with the enormous intellectual skills you possess, these must be leveraged by making sound and informed decisions about your future. Take a long-term perspective by thinking deeply about what success would look like for you in 10–15 years, and what big problem you hope to solve in biomedicine (see Box 1). Too often, young scientists focus only on their next step (or next NIH grant) and do not take a long view. You might consider additional training, such as in growing areas of artificial intelligence and “big data” or by obtaining one of the newer Master of Business Administration (“tech MBA”) degrees that combine traditional management and leadership skills with specialized information relevant to the technology industry [7].

**Box 1: Questions for planning your career in biosciences**

- What problem in life do I hope to solve?
- What does success look like for me in the next 10–15 years?
- How do I draw a roadmap to get from where I am today to that 10–15-year time horizon?
- What other training or “stackable credentials” (e.g., certificates, degrees, executive leadership training) do I need?
- Is there a role model for someone who has achieved something similar? Would that person be willing to provide mentorship?
- Is it fun and energizing (it’s supposed to be) or does it make me anxious and upset (then don’t do it)?

For the second point, remember that this is your life and future; it should be fun and exciting. If you find that your decisions make you anxious or fretful, chances are it’s not right for you. Your career path and trajectory should be energizing or even whimsical, meaning playful or imaginative. It should also align with efforts to cultivate your own personal brand (as I have written about in this journal previously) [8]. Fragmentation or unreliability in governmental biomedical funding could also make it tougher to remain at one company or institution for your entire career. Most likely you may need to switch employment, cities, or in some cases even countries to solve your big problem in life or meet your own personal expectations for success over a 10–15-year (or longer) horizon. Your personal brand moves with you, and that too should be a source of career satisfaction and doing good in the world.

## Abbreviation

**:**
NIH: National Institute of Health
